# Influence of the Al Content on the Properties of Mechanically Alloyed CoCrFeNiMn_X_Al_20−X_ High-Entropy Alloys

**DOI:** 10.3390/ma15227899

**Published:** 2022-11-09

**Authors:** Hana Thürlová, Filip Průša

**Affiliations:** Department of Metals and Corrosion Engineering, University of Chemistry and Technology Prague, Technická 5, 166 28 Prague, Czech Republic

**Keywords:** mechanical alloying, high-entropy alloys, microstructure, mechanical properties, high-temperature resistance, high-temperature oxidation

## Abstract

The equiatomic CoCrFeNiMn alloy prepared by mechanical alloying and spark plasma sintering underwent partial substitution of Mn by Al (5, 10 and 15 at.%) to determine its influence on mechanical properties and thermal stability. It was discovered that the higher the Al content, the higher the volume fraction of the hard phase with primitive cubic (PC) crystallographic lattice, which increases the hardness and strength of the alloys. The most promising mechanical properties have been achieved in the CoCrFeNiMn5Al15 alloy reaching the compressive yield strength (CYS) of 2135 ± 21 MPa and the ultimate compressive strength (UCS) of 2496 ± 21 MPa. All the prepared alloys showed good thermal stability as they maintained or only slightly reduced their initial hardness during the 100 h annealing at 800 °C. Furthermore, the higher the Al content, the higher the resistance against high-temperature oxidation. The oxidic layer changed its composition from Mn-oxides (CoCrFeNiMn15Al15 alloy) to Al-based oxides with exceptional protective properties.

## 1. Introduction

The high-entropy alloys nowadays belong to modern metallic materials, which were discovered in 2004 [1], and since then, their importance is steadily increasing. According to the definition, they are at least composed of four elements with their content ranging from 5–35 at.% [2,3,4]. Although they could be considered a relatively new material, it is already evident that their application potential is enormous. Their properties are often quite diverse and non-dependent on the actual chemical composition, but also the processing technique used for their production.

The alloys are prepared usually by conventional methods such as casting [5], which is performed in cooperation with induction melting [6] or arc melting [7,8]. Such prepared alloys then show a dendritic microstructure [7,9], which yields the lowest resulting mechanical properties compared to well-refined ultrafine-grained or even nanostructured materials prepared by the most modern methods. A very popular additive printing [10,11,12,13], allowing for the creation of complex shaped materials, uses a high-energy source locally melting the material. This results in a refined microstructure, although the main problem is the residual porosity that deteriorates the mechanical properties of such materials. Therefore, the powder metallurgy techniques [14,15,16,17] seem to be the most perspective processing techniques able to produce nearly full-density compact materials with ultrafine-grained and a homogeneous microstructure [18]. The combination of mechanical alloying (MA) [9,19,20] and compaction technique, e.g., spark plasma sintering (SPS) [21,22], is the most pronounced approach when it comes to achieving the highest strengths and hardnesses of these materials.

The first high-entropy alloy, reported by the British scientist Cantor [1], was the equiatomic CoCrFeNiMn alloy. It was proved that the chosen preparation technique has a significant influence on the properties of HEAs, mainly due to the large number of publications related to this alloy. While a dendritic structure was observed in the alloy prepared by arc melting [1], the alloy prepared by a combination of MA + SPS [19,20,23] was composed of only a solid solution with FCC crystallographic lattice. Thus, this positively affected the properties of the alloy, mainly increasing its strength [22]. In the work of Průša et al. [19], the CoCrFeNiMn equiatomic alloy reached a strength of 1610 MPa while having a hardness of 352 HV. On the other hand, the same alloy prepared by arc melting reached a strength of only 491 MPa, having a hardness of 170 HV [24]. Among the variety of properties influenced by the chosen preparation technique, this alloy is also well known for its ductility, reaching for the MA + SPS alloy up to 34% [19]. This behavior is beneficial due to the further processing or the fact that these alloys can be further strain-hardened.

The CoCrFeNiAl alloy is another important alloy from a broad family of HEAs. Thus, when prepared by mechanical alloying, it is composed of two solid solutions having FCC and BCC crystallographic lattices [17,25]. During the consequential compaction, the BCC solid solution underwent a partial phase transformation creating a tetragonal σ phase, which is usually depleted in Al [17]. Therefore, the phase composition has a significant influence on its mechanical properties. Compared with the CoCrFeNiMn alloy, the CoCrFeNiAl alloy reaches nearly twice as higher hardnesses reaching up to 625 HV [26]. Furthermore, the strength of this alloy is higher (1907 MPa), although at the expense of ductility due to the presence of BCC solid solution [26].

The senary CoCrFeNiMn_x_Al_20−x_ alloys were prepared to describe the optimal relation between the hardness, strength and ductility according to the amount of Al. Thus, the partial substitution of Mn by Al and its influence on the phase composition, mechanical properties and high-temperature oxidation were described. It was discovered that the increasing content of Al resulted during the SPS compaction in the phase transformation of the BCC phase into primitive cubic (PC), while the oxidation resistance significantly improved.

## 2. Materials and Methods

### 2.1. Preparation of Alloys

The CoCrFeNiMn15Al5, CoCrFeNiMn10Al10 and CoCrFeNiMn5Al15, further denoted as Mn_X_Al_20−X_, were prepared by the powder metallurgy techniques. For this purpose, a MA of the powder mixture made of an appropriate amount of each element was used, whose basic characteristics are summarized in Table 1. The MA was performed in a planetary mill Retsch PM 100 (Haan, Germany) under an Ar atmosphere to prevent any undesirable oxidation. A jar and milling balls, both made of AISI 420 stainless steel, were used. The weight ratio of the milling balls to the weight of the prepared powder was 15:1. As a process control agent (PCA), which reduces the excessive cold welding of the powder and milling elements/walls, an addition of 4 wt.% of n-heptane was used. The alloying was performed at 400 rpm with a 10 min pause after 30 min segments of the alloys to cool down the powder mixture. Therefore, the entire process took 10 h and 30 min, meaning the alloy underwent a total of 8 h of alloying.

The alloys prepared by the mechanical alloying were further compacted using the SPS (FCT Systeme, HP D 10, Rauenstein, Germany), during which the 10 g of the prepared alloy was in the Ar atmosphere heated up at 200 °C/min until reaching the compaction temperature of 1000 °C. The powder then was compressed within 1 min by a pressure of 48 MPa and remained compressed for another 9 min and then was cooled down with the maximal cooling speed of the device, which varies according to the temperature gradient between the graphite mold and the water-cooled pistons. The achieved cooling rate can be described by a polynomial Equation (1):

(1) $$v=-1\cdot 10^{-9}t^{5}+8\cdot 10^{-6}t^{4}-0.0209t^{3}+26.196t^{2}-16421t+4\cdot 10^{6}$$

where *t* stands for time (s). This combination of compaction conditions is favorable to suppressing any deleterious microstructural coarsening, thus retaining the excellent properties of such materials.

### 2.2. Characterization of the Prepared Alloys

The chemical and phase composition was determined using the X-ray fluorescence (XRF, ARL XP 2400, Almelo, The Netherlands) analysis and X-ray diffraction (XRD, PANalytical X’Pert Pro, MPD, CoKα1, λ = 1.78901 × 10^−10^ m, Almelo, The Netherlands). The microstructure of the prepared powders and compacts were observed on the metallographic cross-sections, which were ground on SiC abrasive papers and polished on polycrystalline diamond suspensions with particle sizes of 3 and 1 μm. Furthermore, the cross-sections were etched in aqua regia (HNO_3_ and HCl mixed in a ratio of 3:1) diluted with distilled H_2_O in a ratio of 1:1. The microstructure was observed using light microscopy (LM, Nikon Eclipse MA 200, Yokohama, Japan) and scanning electron microscopy (SEM, Tescan Lyra 3, Brno, Czechia) equipped with an energy dispersion spectrometer (EDS Oxford Instruments, 80 mm^2^, High Wycombe, UK). The working parameters of the SEM were composed of WD: 9.00 mm; BI: 11.00; HV: 15 kV; BSE/SE detectors mixed in a ratio of 80:20. The LM micrographs were used to determine the average surface porosity using the threshold method in ImageJ 1.52a software (Bethesda, MD, USA), and these results were compared with the relative density of the samples determined using Archimedes principle (glycerine tempered at 20 °C). On the other hand, the SEM micrographs were used to determine the grain size of the present phases using the Feret diameter measurements.

The compacts were examined using the Future-Tech FM-700 (Kawasaki-City, Japan) hardness measuring device for Vickers hardness using a 1 kg load with a dwell time of 10 s. The measured values were accompanied by the confidence interval with a level of significance α = 0.05. Further, the mechanical properties of the alloys were determined using the compressive stress–strain testing performed on the universal testing device LabTest 5.250SP1-VM (Opava, Czechia) with a strain speed of 0.001 s^−1^. For this purpose, quadrangular prismatic samples, having a height of 1.5× the size of the base, were used. Typically, the w × d × h dimensions of the samples varied around 3 mm × 3 mm × 4.5 mm. The mechanical properties were measured according to the standard DIN 50 106.

The quadrangular prismatic samples were compressively tested at laboratory temperature either in their as-compacted state and also after being annealed at 800 °C for 100 h. Their thermal stability was determined using the hardness measurements during the annealing at intervals of 1; 2.5; 5; 7.5; 10; 20; 40; 60; 80 and 100 h, after which they were cooled down outside the electric resistance furnace and ground on P800 SiC abrasive paper. In the same time intervals, oxidation kinetic was determined by measuring the weight gains of the sample. The annealed samples were also investigated for microstructural changes after 100 h of annealing. In addition, the oxide layer was characterized by the SEM + EDS line scan analysis to describe the present oxides.

## 3. Results and Discussion

### 3.1. Composition and Microstructure

#### 3.1.1. Chemical and Phase Composition

In the frame of this work, three alloys with chemical composition confirmed by the XRF analysis, whose results are shown in Table 2, were prepared. The values of element concentration correspond partially to the intended compositions, although some elements showed slightly higher content, especially Ni. The deviations from the desired composition were caused by the different behavior of the pure elements at the early stages of the mechanical alloying. Especially the Co and Cr tend to stick on the walls and corners of the milling jar, decreasing their content within the alloy. On the other hand, the content of Fe and Ni increased in the later stages of the alloying due to the sharp hardness change of the newly formed alloys causing contamination of the prepared alloys with the material of the milling jar. In addition, all the alloys showed the presence of some impurities, which reached up to 2.3 at.% in the Mn5Al15 alloy, mainly composed of Si (2.0 at.%), Ti (<0.1 at.%), V (<0.1 at.%) and other elements.

**Figure 1:**
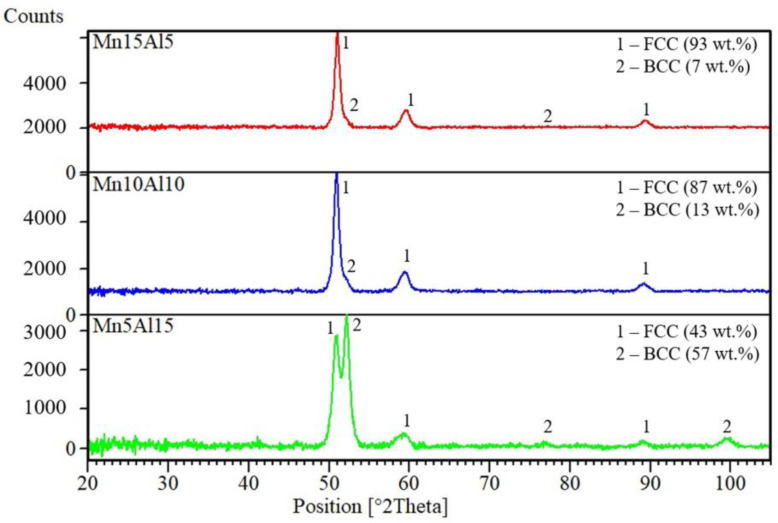
XRD patterns of the MA powder alloys corresponding to their chemical compositions.

Partial substitution of Mn by Al leads to a change of the phase composition of the prepared powder alloys. As is shown in Figure 1, the present phases were composed of two solid solutions with FCC and BCC crystallographic lattices, matching the reference cards for the Cr_0.293_Fe_0.468_Co_0.239_ (JCPD card no. 04-021-2007) and Ni_0.4_Al_0.6_ (JCPD card no. 04-001-2613), respectively. The ratio between the FCC and BCC solid solutions changed as the content of Al increased, stabilizing the BCC solid solution. This documents the 7 wt.% of BCC found in the Mn15Al5 alloy, which further increases until reaching up to 57 wt.% (Mn5Al15 alloy). The observed behavior corresponds to the research of Mohanty et al. [17] reporting the presence of BCC solid solution in their equiatomic quinary CoCrFeNiAl alloy, or with the work of Wang et al. [27] describing the phase composition of equiatomic alloy CoCrFeNiMnAl. Moreover, the content of the hard and yet more brittle BCC phase can be influenced by the content of the Al within the alloy, becoming a powerful tool to precisely tailor the targeted properties.

**Figure 2:**
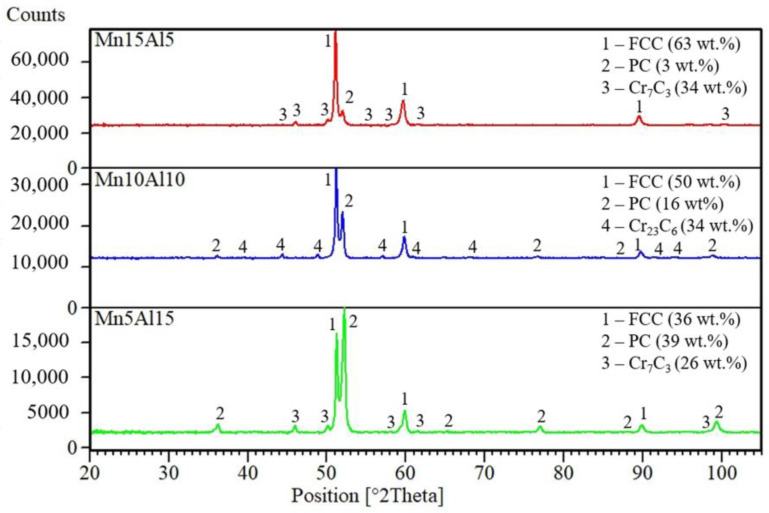
XRD patterns of the MA + SPS compact alloys corresponding to their chemical compositions.

During the SPS compaction, a phase transformation occurred as is shown in Figure 2. The present BCC solid solution completely transformed into a phase with a primitive cubic (PC) lattice, which matches the AlNi phase (JCPD card no. 01-073-2594). Similar findings were reported also in the works focused either on quinary CoCrFeNiAl [17], or senary CoCrFeNiMnAl [28] alloys, although the newly formed phase was reported to have a tetragonal lattice. The difference can be found in the mechanical alloying process, which can be considered as a non-equilibria preparation technique producing metastable phases, which further transforms into more stable ones. In addition, the SPS compaction allowed the formation of carbides identified as Cr_7_C_3_ (Cr_23_C_6_ in the Mn10Al10 alloy) within the microstructure. There are two reasons for their formation: firstly, the preparation is performed in an environment rich in C, allowing its supersaturation; and secondly, the compaction is performed in a graphite die, which serves as another source of C.

#### 3.1.2. Microstructure

**Figure 3:**
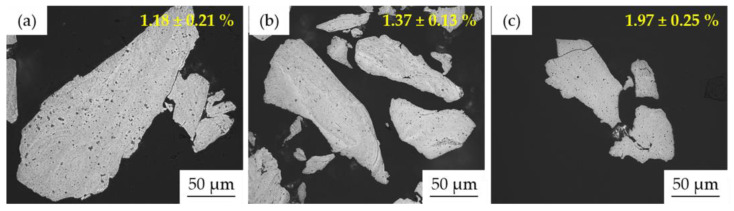
LM micrographs of the MA powder alloys with marked surface porosity determined using the threshold method: (**a**) Mn15Al5, (**b**) Mn10Al10, (**c**) Mn5Al15.

The microstructure of prepared powder alloys was observed using light microscopy (LM) and also scanning electron microscopy (SEM). The corresponding LM micrographs are shown in Figure 3, accompanied by the average surface porosity values determined on the non-etched samples using the threshold method. It was discovered that the higher the content of Al within the alloy, the higher the surface (also internal) porosity of the prepared powders. This directly points out the increasing hardness of the materials and the reduction in their ductility. As one can see, the microstructure of all the MA powder alloys was ultrafine-grained and homogeneous. Considering the SEM + EDS element distribution maps (not shown), showing the uniform element distributions, the present lamellas were highly likely a consequence of etching, which preferentially dissolves certain crystallographic planes manifesting themselves by such appearance.

**Figure 4:**
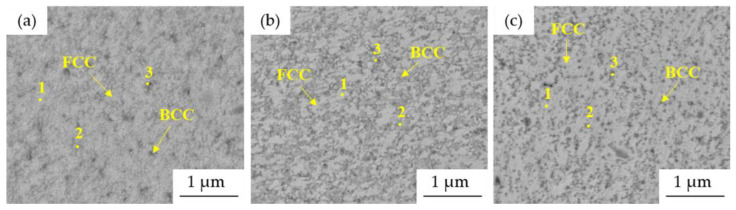
SEM micrographs and points of EDS analysis of MA powder alloys: (**a**) Mn15Al5, (**b**) Mn10Al10, (**c**) Mn5Al15 (BSE + SE, MAG: 50.0 kx, BI: 11.00, WD 9.00 mm).

The more detailed SEM micrographs (Figure 4) confirmed the ultrafine-grained and homogeneous microstructure of the prepared powders. The microstructure can be characterized by the presence of two phases that can be distinguished by their color as follows: FCC solid solution (bright areas), BCC solid solutions (gray areas) and some pores, which manifested themselves as dark gray areas.

**Figure 5:**
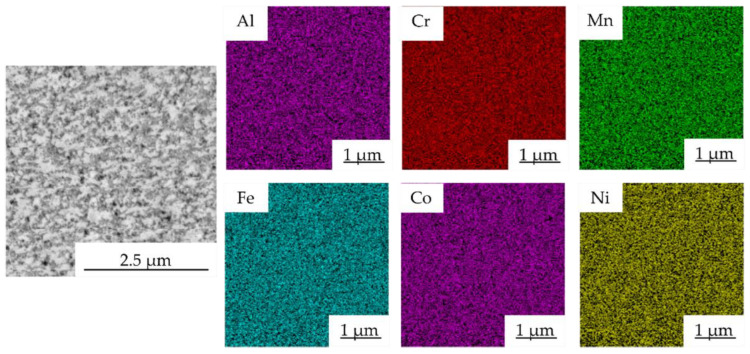
SEM + EDS map of element distribution in Mn10Al10 MA powder alloy.

The chemical composition of each structural constituent, marked with numbers in Figure 4, was determined by the SEM + EDS point analysis, and its results are summarized in Table 3. According to the results, the element content seems uniform, although some minor differences in Al content are visible. The bright phases, identified as the FCC solid solution, were depleted of Al (points marked as 1 in Figure 4), while the gray areas were enriched with it (points marked as 2 in Figure 4). This corresponds to the findings of others, reporting the stabilization of BCC solid solutions due to the presence of Al [17,28]. Nevertheless, the differences in the chemical composition of each phase are strongly affected by their ultra-fine nature considering the surrounding area and the interaction volume. This can be confirmed by the SEM + EDS element distribution maps of the Mn10Al10 alloy (Figure 5), showing the uniform distribution of elements within the present phases, a similar finding found in the remaining powder alloys.

**Figure 6:**
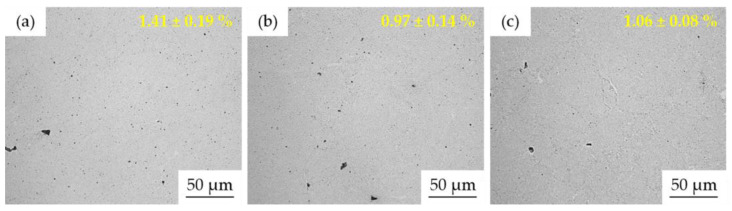
LM micrographs of the MA + SPS alloys with marked surface porosity determined using threshold method: (**a**) Mn15Al5, (**b**) Mn10Al10, (**c**) Mn5Al15.

According to the previous approach, the microstructure of the compact MA + SPS samples observed using LM is shown in Figure 6. The values of the average surface porosity (shown in Figure 6) confirm that the SPS compaction reduced the previously observed internal porosity of the individual powder particles down to approximately 1% for the Mn10Al10 and Mn5Al15 alloys. On the other hand, the porosity of the MA + SPS Mn15Al5 alloy was a bit higher, reaching up to 1.41 ± 0.19%. These results are in good agreement with the relative density determined using Archimedes principle, corresponding to the increasing content of Al within the alloy as 98.5, 98.7, and 98.9%.

Accordingly, the microstructure appearance changed, retaining its ultrafine-grained character, while the previously lamellar morphology seen in the powder alloys (see Figure 3) completely disappeared. This corresponds to the previously mentioned presumption based on the preferential dissolution of certain crystallographic orientations within the powder particles. Since the MA is a sort of metastable process, the consequential SPS compaction at a temperature of 1000 °C is responsible for the disappearance of the previously seen lamellae.

**Figure 7:**
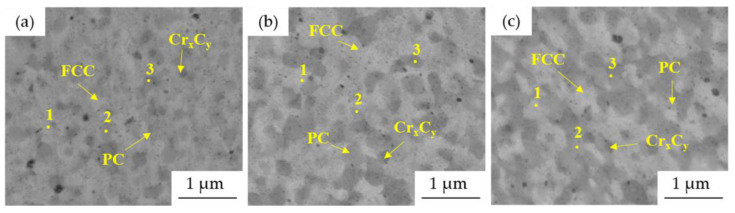
SEM micrographs and points of EDS analysis of MA + SPS compact alloys: (**a**) Mn15Al5, (**b**) Mn10Al10, (**c**) Mn5Al15 (BSE + SE, MAG: 50.0 kx, BI: 11.00, WD 9.00 mm).

The more detailed microstructures of MA + SPS (Figure 7) alloys observed using SEM proved the ultrafine-grained microstructure of the materials. The size of the present phases did not exceed 1 μm in each of the investigated alloys, although when compared to Figure 4, it showed certain coarsening. The results of the SEM + EDS point analysis of the phases marked in Figure 7 are summarized and shown in Table 4. According to these results, the compaction leads to the formation of phases enriched in Al and Ni (medium gray areas, points no. 2) and phases enriched in Cr (dark gray areas, points no. 3). This confirms the XRD results (Figure 1 and Figure 2) reporting the BCC⟶PC phase transformation and formation of the Cr-based carbides within the MA + SPS alloys. The BCC fully transformed into a new phase with PC crystallographic lattice whose content was increasing with the content of Al within the alloy, a phenomenon reported in [17,28]. On the other hand, the Cr-based carbides were enriched in all of the alloy elements suggesting a strong element substitution within them.

**Figure 8:**
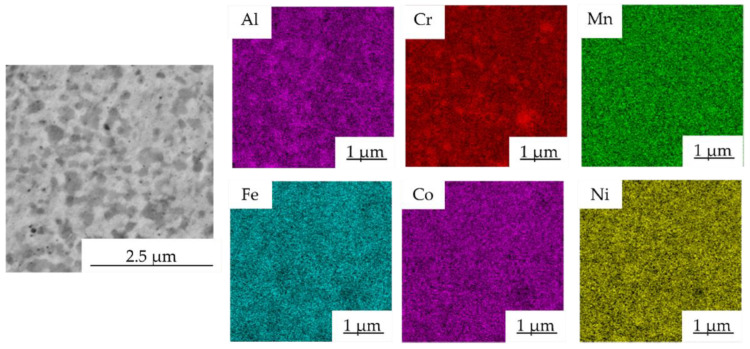
SEM + EDS map of element distribution in the Mn10Al10 MA + SPS compact alloy.

The presence of phases with different content of Al, Ni and Cr was confirmed by the SEM + EDS element distribution maps of the Mn10Al10 as shown in Figure 8. The FCC phase (bright gray areas, points no. 1) showed the highest content of Fe, starting at 23.0 ± 0.3 at.% for the Mn15Al5 alloy and reaching up to 28.5 ± 0.5 at.% for the Mn5Al15 alloy (Table 4). Furthermore, the FCC phase retained the content of Cr and Co across all the MA + SPS alloys, while the Ni and Mn content decreased at the expense of increasing the Al content within this phase. On the other hand, the present PC phase (gray areas) showed quite similar behavior with only a small change in the Ni content, which reached its maxima in the Mn10Al10 alloy and then slightly decreased. However, this PC phase showed the strongest enrichment of Al within it among all the observed structural constituents, suggesting that Al stabilizes it. The present carbides (Cr_7_C_3_ or Cr_23_C_6_), except for the Al content, showed a similar distribution of other elements confirming the above-mentioned results of SEM + EDS point analysis.

### 3.2. Mechanical Properties

**Figure 9:**
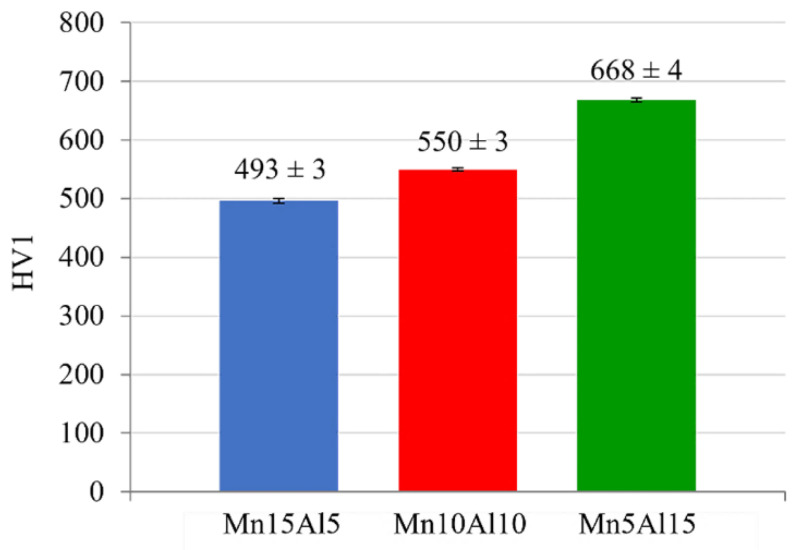
Vickers hardness of the MA + SPS compact alloys. (Error bars represent confidence intervals).

The prepared MA + SPS compacts were investigated for the influence of partial substitution of Mn by Al on the resulting mechanical properties. According to the Vickers hardness measurements, which results are presented in Figure 9, the prepared alloys showed a strong dependence of their hardness on the actual chemical composition. Increasing the content of Al resulted in an increase in the hardness from starting at 493 ± 3 HV 1 (Mn15Al5) and going up to 668 ± 4 HV 1 (Mn5Al15). This value is even higher than that of the quinary equiatomic CoCrFeNiAl alloy prepared using the same techniques reaching only 646 HV [26]. This illustrates the significant influence of the PC phase and carbides whose mutual fraction increased within the compacts with the content of Al. More importantly, the hardness of the Mn5Al15 alloy was significantly higher than the 352 HV of the equiatomic quinary single-phased CoCrFeNiMn alloy prepared using the same experimental setup reported in [19]. Therefore, partial substitution of Mn by only 5 at.% of Al results in a humongous hardness increase equal to 141 HV and further increases with the content of Al.

**Figure 10:**
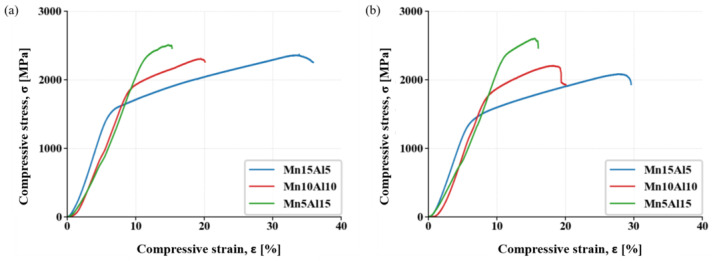
Compressive stress–strain test for alloys at room temperature: (**a**) MA + SPS; (**b**) MA + SPS after annealing (800 °C, 100 h).

The prepared compacts were also investigated using compressive stress–strain testing at laboratory temperature after compaction. It was discovered that with increasing the content of Al, the compressive yield strength (CYS) and ultimate compressive strength (UCS) values increased (Figure 10a). However, the observed strengthening was accompanied by the rising brittleness of the tested alloys that can be directly matched to the weight fractions of the present phases (see Figure 2). One can see that increasing the content of Al at the expense of Mn stabilizes and further increases the weight fraction of the AlNi phase with the PC crystallographic lattice at the expense of the ductile FCC solid solution. In addition, the weight fraction of the carbides remains the same or even decreases, suggesting that the AlNi phase is the main reason for the increasing brittleness. However, considering the increasing content of Al within the FCC solution reaching up to 10.1 ± 1.0 at.%, the solute strengthening via Al should also be taken into account. This presumption corresponds to the work of Varvenne et al. [29], who reported positive strengthening effects of the Al solutes added to the CoCrFeNi alloy, and also with work of Wang et al. [30], who showed that Al addition in Al_x_CoCrFeNi alloys is increasing mechanical properties. Accordingly, the Mn5Al15 alloy showed the highest average values of CYS and UCS, reaching up to 2135 ± 21 MPa and 2496 ± 21 MPa, respectively. The strengthening of the alloys was accompanied by ductility reduction, as is shown in Table 5, reaching up to 20% compared to most (Mn15Al5) ductile alloys.

### 3.3. Thermal Stability and High-Temperature Oxidation

**Figure 11:**
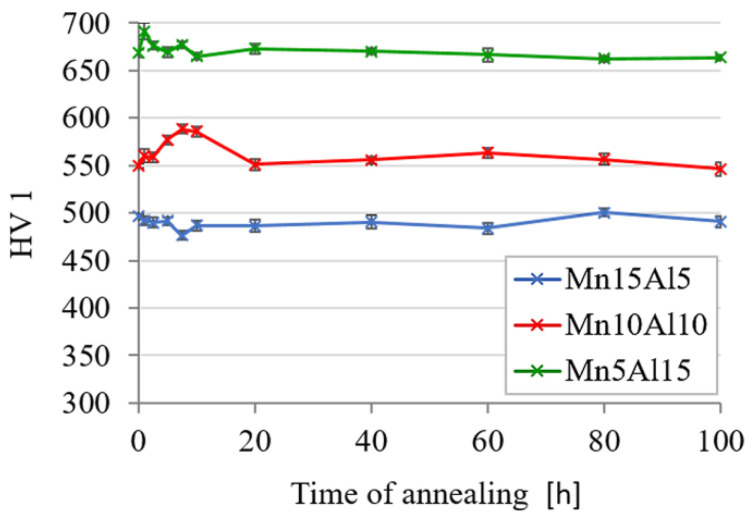
Thermal stability of the MA + SPS alloys as a hardness change during annealing at 800 °C. (Error bars represent confidence intervals).

The thermal stability of the prepared compacts was evaluated as hardness change during the long-term annealing at 800 °C. According to the results shown in Figure 11, all the tested alloys showed exceptional stability since they retained their initial hardnesses. Moreover, the Mn10Al10 alloy showed during the first 10 h of annealing a hardness increase, likely caused by the increasing formation of the carbides and their growth, showing similar behavior to the CoCrFeNiMnAl alloy annealed at a temperature of 1400 K (1126.85 °C) as reported in [28].

In addition, the samples were also compressively tested at laboratory temperature after being annealed for 100 h at 800 °C (Figure 10b, Table 5). It was found that Al positively affects the thermal stability of the alloys. The compacts containing up to 10 at.% of Al softened, reducing their CYS and UCS, which was especially significant in the Mn15Al5. Increasing the content of Al up to 10 at.% (Mn10Al10 alloy) resulted in a less significant reduction in the mechanical properties. However, increasing the content of Al at 15 at.%, the average CYS and UCS values increased or remained the same when accounting for the confidence interval of the measurements. Therefore, the Mn5Al15 alloy reached the highest CYS and UCS of 2214 ± 84 MPa and 2570 ± 89 MPa, respectively, among all the tested MA + SPS alloys, even slightly outperforming the properties of the same alloy before being annealed. This was achieved due to the high concentration of the carbides forming an effective barrier hindering the dislocation movement.

**Figure 12:**
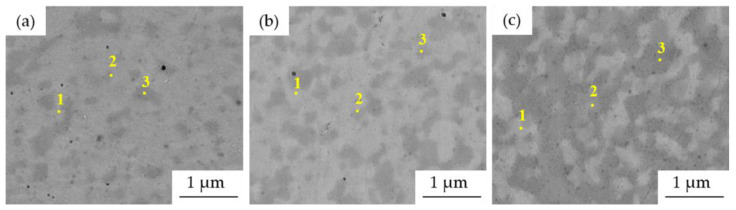
SEM micrographs and points of EDS analysis of MA + SPS compact alloys after 100 h annealing at 800 °C: (**a**) Mn15Al5, (**b**) Mn10Al10, (**c**) Mn5Al15 (BSE + SE, MAG: 50.0 kx, BI: 13.00, WD 9.00 mm).

According to the change in the properties of the tested alloys, the microstructural-related effects associated with the accelerated diffusion processes are observed at elevated temperatures (Figure 12). As one can see, the appearance of the MA + SPS alloys did not undergo any extremely significant change compared to the previously shown micrographs of the MA + SPS compacts (see Figure 7). The grain coarsening is the most pronounced effect related to this behavior, which is happening in most metallic materials. However, the HEAs are known to exhibit extremely low diffusion coefficients [4] within the alloys, which suppresses the microstructural coarsening even when exposed at such high temperatures as 800 °C. The grain size of the FCC phase increased by 7–14% during annealing (Table 6), still retaining a ultrafine-grained microstructure. In comparison, the remaining structural constituents including the Cr_7_C_3_ (Cr_23_C_6_ in Mn10Al10 alloy) and the PC phases showed exceptional thermal stability, retaining their original dimensions after being annealed for 100 h at 800 °C.

In addition, the phase composition of the alloys did not change through annealing, remaining almost identical to those previously reported for the as-compacted samples. The results of the SEM + EDS analysis of the chemical composition of the points marked in Figure 12 are summarized in Table 7. As one can see, the chemical composition of individual phases did not change significantly compared to the results mentioned in Table 4, confirming the presence of the FCC, PC and carbide phases. Therefore, apart from the microstructural coarsening, the phases’ chemical compositions remained the same.

**Figure 13:**
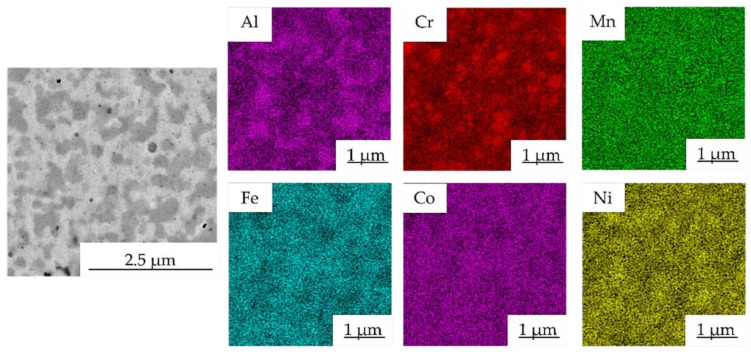
SEM + EDS map of element distribution in the Mn10Al10 MA + SPS compact alloy after 100 h annealing at 800 °C.

The SEM + EDS element distribution maps in the Mn10Al10 alloy are shown in Figure 13 and, according to the results of SEM + EDS point analysis, proved the presence of areas enriched in Cr and areas with a higher concentration of Al. When compared with the element maps of the as-compacted alloy (Figure 8), both areas seem to become coarser, supporting the above-mentioned grain coarsening. Moreover, it suggests the even coarsening of each structural component during the annealing.

**Figure 14:**
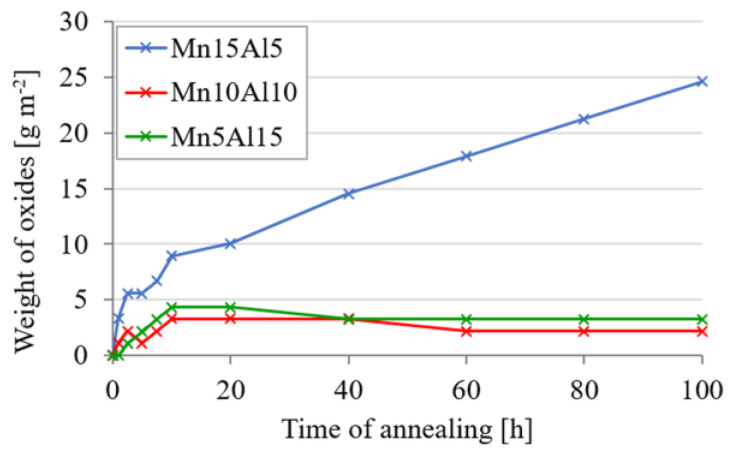
Weight increment of oxides during annealing at 800 °C for 100 h. (Error bars represent confidence intervals).

The high-temperature oxidation at 800 °C was evaluated based on the weight gains of the initially weighted sample with a defined surface. As one can see in Figure 14, the Mn15Al5 alloy showed a sort of parabolic kinetics of oxidation, which is known for rapid weight gain in a matter of minutes and slowing down over time due to the diffusion-driven character of oxygen transport through the developing oxide layer. However, as the annealing prolonged, the oxidation kinetics showed rather a linear mechanism according to the weight gains, suggesting the poor protective behavior of such layer due to, e.g., cracking or formation of pores within the layer. On the other hand, increasing the Al content resulted in logarithmic kinetics of oxidation, suggesting the creation of a dense protective layer, which did not tend to chip off over the time of annealing. Both the Mn10Al10 and Mn5Al15 alloys gained less than 5 g m^−2^ of newly formed oxides after 100 h annealing at 800 °C. Similar behavior was also reported in the work of Lyu et al. [31], who prepared the CoCrFeNiAlx (x = 0.1, 0.5, 1) alloy using vacuum arc melting, which significantly improved its oxidation resistance from 2 at.% of Al.

**Figure 15:**
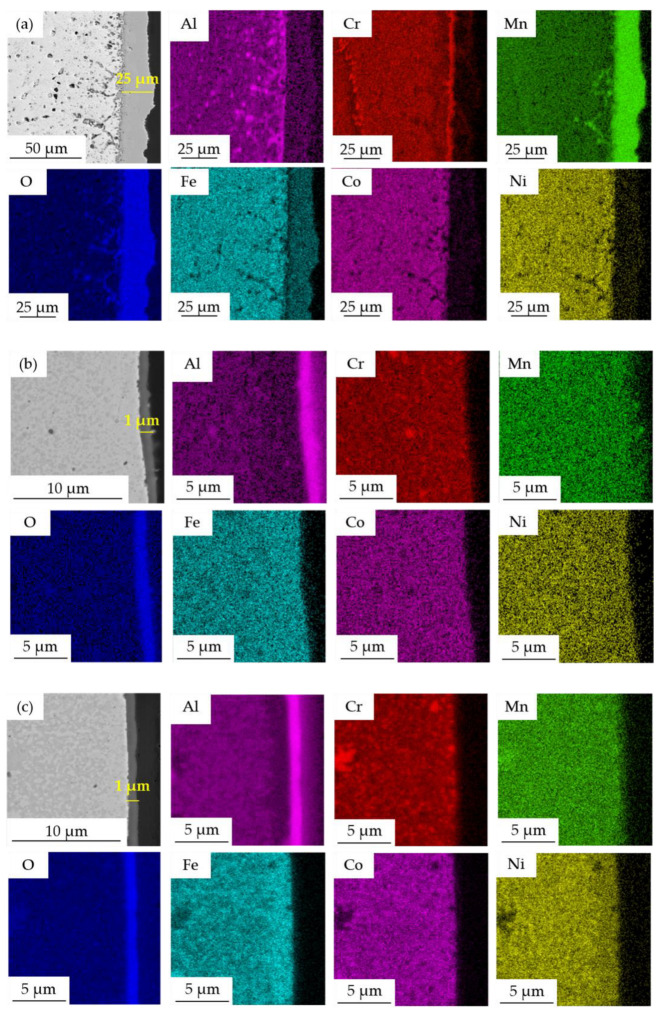
SEM + EDS element distribution maps across the oxide layers formed after 100 h annealing at 800 °C: (**a**) Mn15Al5, (**b**) Mn10Al10, (**c**) Mn5Al15.

The differences in the oxidation kinetics pointed out the importance of the description of the formed oxide layers. Therefore, the metallographic cross-sections of the oxidic layers were prepared and observed using SEM + EDS (Figure 15). In the case of the Mn15Al5 alloy, the outer part of the oxide layer was mainly composed of Mn, suggesting the presence of Mn_x_O_y_ oxides followed by a thin layer of Cr-based oxides found at the metal/oxide interface. Moreover, a significant level of porosity was found within its closest proximity of it. The overall thickness of the whole layer after 100 h annealing reached approximately 25 µm. More importantly, the Mn15Al5 alloy was the only one showing strong sub-surface oxidation and developed internal porosity due to the diffusion of Mn toward the oxide layer. It was found that the sub-surface porosity reached up to ≈100 µm into the alloy core and likely may further progress with the increasing time of annealing. The present oxides within the metal were mainly composed of Mn and Al. Increasing the content of Al above 5 at.% resulted in a completely different behavior, forming a well-protective oxide layer containing foremostly Al, suggesting the formation of Al_x_O_y_ without any traces of chipping or formation of sub-surface oxides. The thickness of these layers was in both the remaining alloys ≈ 1 µm. In addition, the oxide layer did not contain any interlayers at the interface of the metal/oxide as did the Mn15Al5, which effectively hindered the diffusion processes. Such behavior manifested itself as swift growth of the thin layer, which then completely suppressed the ongoing oxidation.

**Figure 16:**
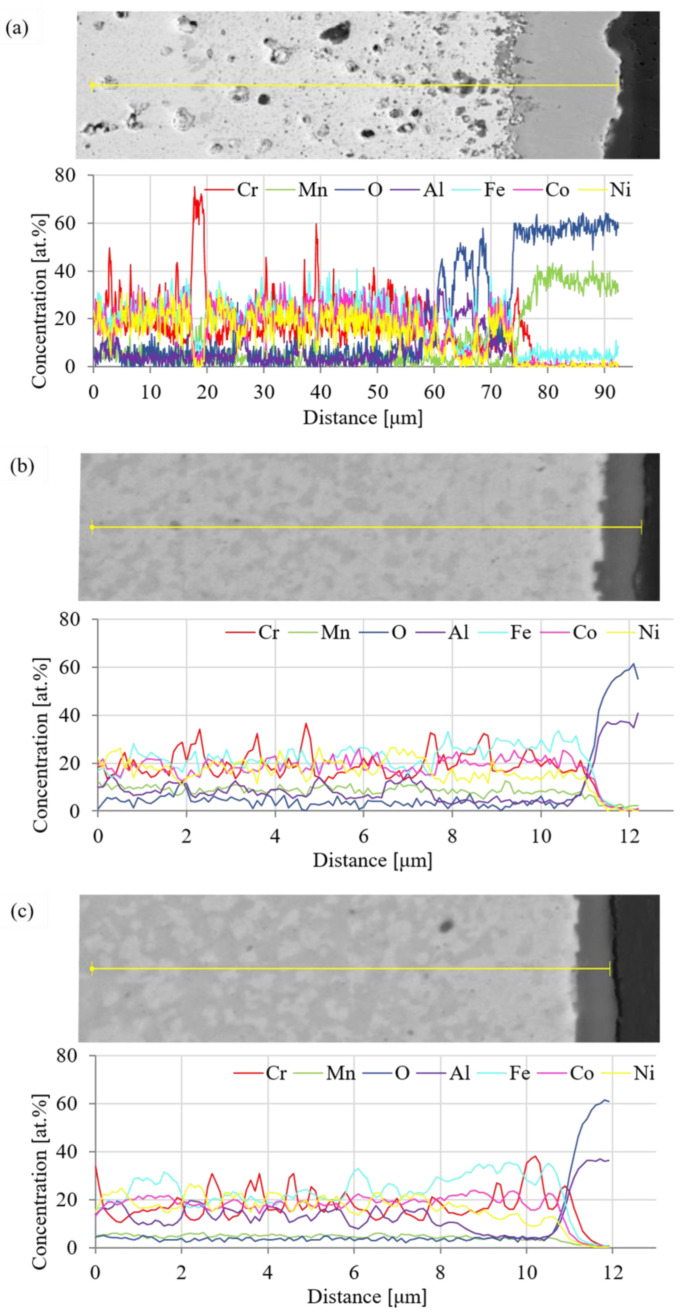
Quantified SEM + EDS line analysis (yellow line) across the oxide layers formed after 100 h annealing at 800 °C: (**a**) Mn15Al5, (**b**) Mn10Al10, (**c**) Mn5Al15.

The present oxide layers were also characterized by SEM + EDS line analysis with quantification of the results, as shown in Figure 16. These analyses confirmed the above-made presumptions, e.g., that the oxide layer of the Mn15Al5 alloy is mainly composed of one type of Mn-based oxide accompanied by an increased content Fe within it, which is highly likely to form a complex oxide. As the line analysis moves closer to the metal/oxide interface, the content of Cr steeply increases and is accompanied by O, confirming the above-made presumption based on the formation of a Cr-based oxide interlayer. In comparison, the remaining MA + SPS showed the presence mainly of Al within the oxide layer without any traces of the formation of sub-layers on the metal/oxide interface. Both the alloys showed a certain depletion of Al reaching ≈2.5 µm deep into the alloy.

## 4. Conclusions

The CoCrFeNiMn alloy underwent a partial substitution of Mn by Al forming three distinctive alloys—CoCrFeNiMn15Al5, CoCrFeNiMn10Al10 and CoCrFeNiMn5Al15. It was found that the content of Al within the alloy changed the phase composition, the microstructure appearance and foremostly the mechanical properties and oxidation resistance. All the prepared MA + SPS alloys showed overall exceptional properties due to a formation of homogeneously distributed sub-micrometric phases within the microstructure. The composition of the present phases in the powder alloy changed according to the content of Al, stabilizing the BCC solid solutions at the expense of the FCC solid solution. Moreover, when compacted, the BCC solid solution underwent complete phase transformation into a new phase with a PC lattice, which was also accompanied by a formation of Cr-based carbides. According to the content of Al within the alloy, the weight fraction of the PC phase increased, improving the hardness and strength of the alloys. The highest hardness, compressive yield and ultimate compressive strengths were achieved in the CoCrFeNiMn5Al15, reaching 668 ± 4 HV, CYS of 2135 ± 21 MPa and UCS of 2496 ± 21 MPa, respectively. Furthermore, this alloy was the sole one that improved its properties when long-term annealed for 100 h at 800 °C. All the alloys showed only a minor microstructural coarsening, which resulted in good thermal stability, e.g., proved by only a negligible change in their hardnesses and only a minor change of the strengths. The oxidation kinetics changed according to the content of Al initially from a parabolic (Mn15Al5) to a logarithmic (Mn10Al10 and Mn5Al15) rate, caused by the formation of a protective layer of Al_2_O_3_ instead of the Mn_x_O_y_ oxidic layer observed in the case of the first-mentioned alloy. Moreover, the sub-surface oxidation and porosity caused by the Mn diffusion toward the oxide layer were completely suppressed from 10 at.% of Al, reducing the overall thickness of the oxide layer from ≈25 μm (Mn15Al5) down to ≈1 μm. Considering the above-stated, changing the content of Al can be used for precise tailoring of the complex performance of the prepared alloys to achieve desired properties not only at the laboratory but also at elevated temperatures.

## Figures and Tables

**Table 1:** Characteristic of element powders.

	Co	Cr	Fe	Ni	Mn	Al
Purity [wt.%]	99.8	99.0	99.0	99.5	99.6	99.7
Particle size [µm]	<2	<44	<9	<10	<10	<50

**Table 2:** Chemical compositions (at.%) of compacted alloys determined by XRF analysis.

Alloy	Element Concentration [at.%]						
	Co	Cr	Fe	Ni	Mn	Al	Others
Mn15Al5	15.6	18.0	21.1	24.9	14.8	4.1	1.5
Mn10Al10	18.6	19.4	21.8	20.7	9.3	8.5	1.7
Mn5Al15	18.4	18.6	23.0	20.8	4.4	12.5	2.3

**Table 3:** SEM + EDS analysis of the chemical composition of points marked in Figure 4.

Alloy	Point	Concentration [at.%]						
		Al	Si	Cr	Mn	Fe	Co	Ni
Mn15Al5	1	4.4 ± 0.1	1.9 ± 0.1	20.4 ± 0.1	14.6 ± 0.2	21.7 ± 0.1	18.4 ± 0.1	18.7 ± 0.1
	2	4.9 ± 0.7	2.7 ± 1.1	19.6 ± 0.3	14.4 ± 0.2	21.2 ± 0.5	18.6 ± 0.4	18.7 ± 0.4
	3	4.1 ± 0.1	1.8 ± 0.4	19.7 ± 0.1	14.8 ± 0.1	21.6 ± 0.2	19.0 ± 0.1	18.9 ± 0.2
Mn10Al10	1	8.7 ± 0.1	1.4 ± 0.1	19.8 ± 0.7	9.9 ± 0.2	21.9 ± 0.5	18.8 ± 0.3	19.5 ± 0.1
	2	9.1 ± 0.4	1.4 ± 0.1	20.1 ± 0.9	9.7 ± 0.1	22.0 ± 0.5	18.8 ± 0.2	18.8 ± 0.4
	3	8.6 ± 0.4	1.4 ± 0.1	19.7 ± 0.9	9.8 ± 0.2	22.4 ± 0.4	19.0 ± 0.3	19.0 ± 0.5
Mn5Al15	1	13.7 ± 0.3	1.9 ± 0.1	19.9 ± 0.8	4.8 ± 0.1	23.0 ± 0.2	18.2 ± 0.1	18.5 ± 0.4
	2	13.7 ± 0.7	1.9 ± 0.1	20.3 ± 1.2	5.0 ± 0.1	22.9 ± 0.1	18.3 ± 0.6	18.1 ± 0.1
	3	13.9 ± 0.8	1.9 ± 0.2	19.1 ± 0.1	4.9 ± 0.2	23.1 ± 0.6	18.6 ± 0.1	18.6 ± 0.1

**Table 4:** SEM + EDS analysis of the chemical composition of points marked in Figure 7.

Alloy	Point	Concentration [at.%]						
		Al	Si	Cr	Mn	Fe	Co	Ni
Mn15Al5	1	4.1 ± 0.1	1.7 ± 0.1	16.7 ± 0.2	14.9 ± 0.1	23.0 ± 0.3	19.8 ± 0.2	19.9 ± 0.4
	2	4.4 ± 0.3	1.7 ± 0.1	15.8 ± 0.5	15.1 ± 0.1	22.8 ± 0.5	19.9 ± 0.6	20.3 ± 0.4
	3	3.2 ± 0.4	1.1 ± 0.1	35.2 ± 6.9	13.4 ± 0.8	19.0 ± 1.4	14.5 ± 1.9	13.5 ± 2.3
Mn10Al10	1	7.7 ± 0.4	1.8 ± 0.2	17.3 ± 1.5	9.9 ± 0.3	24.4 ± 0.9	20.5 ± 0.4	18.5 ± 0.9
	2	7.3 ± 0.8	1.7 ± 0.1	20.4 ± 2.6	9.3 ± 0.2	24.0 ± 2.0	20.0 ± 1.4	17.4 ± 1.1
	3	15.2 ± 0.8	1.7 ± 0.1	15.4 ± 2.8	10.7 ± 0.2	15.7 ± 1.0	16.6 ± 0.6	24.7 ± 1.1
Mn5Al15	1	10.1 ± 1.0	2.9 ± 0.1	17.4 ± 2.0	4.6 ± 0.1	28.5 ± 0.5	20.4 ± 0.6	16.2 ± 0.6
	2	11.4 ± 1.7	1.9 ± 0.1	34.0 ± 2.7	4.8 ± 0.3	19.4 ± 1.3	14.3 ± 0.1	14.2 ± 2.1
	3	16.9 ± 1.8	2.6 ± 0.1	15.4 ± 3.4	5.3 ± 0.2	19.5 ± 1.6	18.3 ± 0.7	22.0 ± 2.1

**Table 5:** Summary of mechanical properties of the MA + SPS compacts in their as-compacted and annealed state (800 °C, 100 h). (± values represent confidence intervals).

Alloy	MA + SPS			MA + SPS after Annealing (800 °C, 100 h)		
	CYS [MPa]	CS [MPa]	Ductility [%]	CYS [MPa]	CS [MPa]	Ductility [%]
Mn15Al5	1458 ± 114	2326 ± 58	35	1284 ± 22	2077 ± 22	29
Mn10Al10	1786 ± 38	2314 ± 80	20	1699 ± 43	2167 ± 71	18
Mn5Al15	2135 ± 21	2496 ± 21	15	2214 ± 84	2570 ± 89	15

**Table 6:** Grain size of the present phases in MA + SPS alloys in their as-prepared state and after being annealed (800 °C, 100 h).

Alloy	Grain Size [nm]					
	MA + SPS			MA + SPS after Annealing (800 °C, 100 h)		
	FCC	PC	Cr_x_C_y_	FCC	PC	Cr_x_C_y_
Mn15Al5	257 ± 38	289 ± 24	228 ± 31	277 ± 43	279 ± 31	213 ± 19
Mn10Al10	268 ± 24	284 ± 39	241 ± 24	312 ± 36	272 ± 36	233 ± 17
Mn5Al15	303 ± 37	300 ± 30	257 ± 33	325 ± 32	309 ± 27	268 ± 19

**Table 7:** SEM + EDS analysis of the chemical composition of points marked in Figure 12.

Alloy	Point	Concentration [at.%]						
		Al	Si	Cr	Mn	Fe	Co	Ni
Mn15Al5	1	5.3 ± 0.5	1.7 ± 0.2	18.8 ± 3.7	11.8 ± 0.3	22.2 ± 0.7	19.4 ± 1.2	20.9 ± 1.4
	2	4.3 ± 0.3	1.8 ± 0.1	16.3 ± 0.8	12.2 ± 0.3	23.4 ± 0.4	20.7 ± 0.6	21.3 ± 0.6
	3	3.4 ± 0.4	1.2 ± 0.1	33.7 ± 4.1	10.6 ± 0.5	19.8 ± 0.9	15.9 ± 1.0	15.5 ± 1.5
Mn10Al10	1	5.8 ± 0.3	2.0 ± 0.2	18.2 ± 1.2	9.0 ± 0.1	26.3 ± 0.8	21.6 ± 0.9	17.2 ± 0.3
	2	8.6 ± 0.9	1.4 ± 0.1	27.6 ± 6.1	9.4 ± 0.4	19.0 ± 1.4	16.0 ± 1.6	17.9 ± 1.8
	3	13.8 ± 1.6	1.6 ± 0.1	13.1 ± 0.8	11.6 ± 0.7	11.6 ± 0.7	16.7 ± 1.3	27.0 ± 2.7
Mn5Al15	1	9.8 ± 2.7	3.0 ± 0.1	14.2 ± 1.1	4.6 ± 0.3	29.8 ± 2.7	21.9 ± 0.5	16.8 ± 4.1
	2	11.5 ± 0.5	2.5 ± 0.1	24.2 ± 1.0	4.6 ± 0.1	22.9 ± 1.1	17.6 ± 0.6	16.7 ± 0.1
	3	17.0 ± 2.4	2.6 ± 0.2	13.0 ± 0.9	5.5 ± 0.4	20.1 ± 3.2	18.8 ± 0.7	23.1 ± 2.4

## Data Availability

Data are contained within the article.
